# Boosting drug development through public-private partnerships – The IMI model

**DOI:** 10.1186/1878-5085-5-S1-A11

**Published:** 2014-02-11

**Authors:** Hugh Laverty

**Affiliations:** 1 IMI, Brussels, Belgium

## 

With a €2 billion budget, the Innovative Medicines Initiative (IMI) is the world’s largest public-private partnership in health. Through its collaborative projects that bring together experts from industry, academia, small and medium-sized enterprises (SMEs), patient groups, and regulators, IMI aims to develop tools and technologies that will speed up the development of safer and better drugs for patients and boost innovation in healthcare. The European Union contributes €1 billion to the IMI research programme through the Seventh Framework Programme (FP7); this is matched by in kind contributions worth at least another €1 billion from the member companies of the European Federation of Pharmaceutical Industries and Associations (EFPIA).

IMI currently has 40 ongoing projects and more are in the pipeline. The projects are delivering excellent results that have an impact on all aspects of the drug development procedure, from adding to our understanding of the underlying biology of diseases, through the identification of potential drugs and testing for safety and efficacy, to the design of clinical trials.

Furthermore, through its projects, IMI is helping to build a more collaborative ecosystem for pharmaceutical research and development (R&D) in Europe. In this environment, IMI projects are addressing obstacles to the transfer of innovation into practice as well as involving relevant groups such as regulators and patients. In executing these collaborative projects, IMI acts as a neutral trusted party, ensuring that intellectual property rights are fairly distributed between partners.

Personalised medicine is a focus of many IMI projects. Disease areas where IMI is working on patient stratification and the tools needed to develop targeted, personalised therapies include rheumatoid arthritis (RA) and related immunoinflammatory conditions, diabetes, severe asthma, and brain disorders such as autism, Alzheimer’s disease, chronic pain, depression, and schizophrenia.

The projects’ approach to patient stratification and personalised medicine can take many forms, including the development of patient reported outcomes, improving the design of clinical trials based upon patient stratification, or using electronic health records to speed up the identification of better healthcare solutions. Furthermore, other IMI projects contribute to this work, especially in the area of knowledge management (as combining data from different sources is not a simple task).

Looking to the future, the draft Strategic Research Agenda of IMI 2 focuses explicitly on personalised medicine – the working title is ‘The right prevention and treatment for the right patient at the right time’.

Ultimately, it is hoped that IMI will provide socio-economic benefits to European citizens, increase Europe's competitiveness globally and establish Europe as the most attractive place for pharmaceutical research and development.

